# Primary Care Physicians’ Perspectives on High-Quality Discharge Summaries

**DOI:** 10.1007/s11606-023-08541-5

**Published:** 2023-11-27

**Authors:** Brittany Chatterton, Jennifer Chen, Eleanor Bimla Schwarz, Jennifer Karlin

**Affiliations:** 1 Department of Internal Medicine, University of California, Davis, Sacramento, CA USA; 2 Center for Healthcare Policy and Research, University of California, Davis, Sacramento, CA USA; 3 Department of Medicine, Division of General Internal Medicine, University of California, San Francisco, San Francisco, CA USA; 4 Department of Family and Community Medicine, University of California, Davis, Sacramento, CA USA

**Keywords:** discharge summaries, transitions of care, primary care physicians

## Abstract

**Background:**

Successful transitions of care require communication between inpatient and outpatient physicians. The discharge summary is the main communication tool used by physicians during these transitions.

**Objective:**

With the goal of improving care transitions, we explored primary care physicians (PCPs) perspectives on characteristics of high-quality discharge summaries.

**Design:**

We conducted semi-structured individual interviews in this qualitative study and surveyed participants for sociodemographic characteristics.

**Participants:**

PCPs were recruited from multiple health systems in California.

**Approach:**

An interview guide was created by the study authors to solicit PCPs’ experiences with discharge summaries and perspectives on four discharge summary templates previously used by large health systems. Interviews were transcribed verbatim and qualitative data were analyzed interactively through thematic analysis.

**Key Results:**

Twenty PCPs participated in interviews lasting an average of 35 min (range 26–47 min). Sixty percent were female. Most (70%) had trained in internal medicine (IM); 5% had trained in both IM and pediatrics and 25% in family medicine. Some (45%) participants practiced both inpatient and outpatient medicine; 55% had exclusively outpatient practices. Half worked in university-affiliated clinics, 15% community clinics, 15% public health clinics, 5% private practice, and 15% multiple clinic types. Many PCPs (65%) had been in practice for ≥ 10 years. Participants reported multiple concerns with typical discharge summaries, including frustration with lengthy documents containing information irrelevant to outpatient care. Suggested recommendations included beginning the discharge summary with action items, clear identification of incidental findings requiring follow-up, specifying reasons for any medication changes, and including dates for treatment regimens rather than expected duration of treatment. Participants highlighted the importance of feedback to trainees to assist in crafting succinct discharge summaries containing relevant information.

**Conclusion:**

Clinical training programs and healthcare systems must optimize discharge summaries for PCPs to achieve goals of providing high-quality care that improves population health.

**Supplementary Information:**

The online version contains supplementary material available at 10.1007/s11606-023-08541-5.

## BACKGROUND

Hospital discharge summaries are a crucial part of patients’ transitions to outpatient care. Traditionally, primary care physicians (PCPs) cared for their own patients in both inpatient and outpatient settings.^1^ Although some physicians and health systems still practice in this model, with the rise of hospitalists in the US healthcare system, most care is now siloed, with different physicians caring for the patient in the clinic and hospital.^2^ The growth of the hospitalist model followed restructuring of hospitals’ Medicare reimbursements from a fee-for-service model to a diagnosis-based reimbursement system.^3^ This change has incentivized reductions in patients’ length of stays in the hospital,^3^ which hospitalists more frequently provided. The need for clear communication during transitions of care has grown in importance as most patients are cared for by different physicians as they transition clinical settings.

Direct communication between inpatient and outpatient physicians is currently infrequent, with studies reporting it occurs only 23–38% of the time.^4, 5^ The majority of communication about care transitions occurs via written discharge summaries.^4–6^ This single written document strives to convey all relevant information for the hospitalization including diagnoses made, treatments rendered, results of testing/laboratory evaluation, and plans for patient follow-up. The Joint Commission has identified six categories of information to include in discharge summaries: reason for hospitalization, significant findings, procedures and treatments provided, patient’s condition at discharge, patient and family instructions, and attending physician signature.^7^ However, specific recommendations on how this information should be expressed and organized are up to the writer’s interpretation. Prior studies have assessed PCPs satisfaction with and availability of discharge summaries^8, 9^ and surveys have identified content that discharge summaries should contain (i.e., a description of hospital course, medications recommended at the time of discharge, follow-up instructions).^10–12^ However, considerable variation remains in the quality of discharge summaries. We therefore sought PCPs’ perspectives on the characteristics of a high-quality discharge summary, with the goal of more successfully bridging care between inpatient and outpatient settings for adult patients.

## METHODS

We purposefully sought the perspectives of PCPs, with experiences working in multiple health systems. We aimed to recruit a diverse group of PCPs based on practice location and settings in Northern California. Physicians were recruited through flyers describing the study distributed to primary care physicians’ clinics and emailing primary care physicians invitations to participate in the study. The email list used for recruitment included physicians who had expressed interest in medical education provided by the UC Davis School of Medicine and included primary care physicians from four major health systems, as well as PCPs working in community-based clinics, and clinics run by the Department of Public Health. Participation was voluntary and compensation was not provided. Inclusion criteria included PCPs caring for adults after completing training in internal medicine, family medicine, or a joint internal medicine/pediatrics residency. Consenting participants completed a brief online survey and then joined a one-on-one semi-structured interview conducted using the ZOOM platform, between March 2021 and November 2021. The interview guide was created by the study authors to assess impressions and use of discharge summaries, patient safety during care transitions, preferred structure of discharge summaries, and content that should be contained within a high-quality discharge summary (Appendix 1). Participants were asked to review and comment on four previously implemented templates which were obtained from four different healthcare systems. The templates were all general templates available within the electronic health record system for writing a discharge summary (Appendix 2). Interviews were audio-recorded and transcribed using natural language processing software. Transcripts were then individually validated against the audio recordings. After completion of data collection, a sample of interviews were evaluated by three research team members (BC, JC, JK) and a coding tree was jointly created to inform our planned thematic analysis.^13–16^ The coding tree was applied to five interviews by BC and JC individually and revised until determined to contain all a priori and emergent themes. After confirming inter-coder reliability of > 80%, all 20 interviews were coded individually by both BC and JC using ATLAS.ti. Discrepancies were adjudicated by JK. Thematic analysis was applied to the codes. This study protocol was approved by the University of California, Davis institutional review board.

## RESULTS

The 20 primary care physicians that participated in this study were affiliated with the University of California, Davis health system clinics (*n* = 10), community clinics including Kaiser Permanente and Sutter Health in Northern California (*n* = 3), Department of Public Health operated clinics (*n* = 3), private practice (*n* = 1), and dual affiliations of a University of California, Davis health system clinic and a Department of Public Health operated clinic (*n* = 3). Most participants were female (60%) and had trained in internal medicine (70%), family medicine (25%), and internal medicine/pediatrics (5%) (Table 1). Participants had considerable clinical experience; 65% had been in clinical practice for 10 or more years. Interviews lasted on average 35 min (range 26–47 min). Many participants wished discharge summary writers would do more to “imagine you’re me!” and provide information relevant to outpatient practice. In other words, participants reported discharge summaries were often written from the perspective of an inpatient care provider rather than written to their audience of PCPs working in the outpatient setting. Additional themes we elicited are outlined in Table 2 with specific recommendations for inpatient physicians who would like to provide PCPs with information that is more easily and efficiently incorporated into outpatient care. These items were identified as enhancements for current discharge summary templates used by inpatient physicians to satisfy the Joint Commission requirements.

**Table 1:** Sociodemographic Characteristics of Californian Primary Care Providers Interviewed About Their Experiences with Hospital Discharge Summaries, 2021

Primary care physicians	*N* (%)
Female	12 (60)
Male	8 (40)
Age	
31–40	7 (35)
41–50	5 (25)
51–60	6 (30)
≥ 61	1 (5)
Declined	1 (5)
Specialty	
Internal medicine (IM)	14 (70)
Family medicine	5 (25)
IM/pediatrics	1 (5)
Years in clinical practice	
< 10 years	7 (35)
≥ 10 years	13 (65)
Practice type	
Exclusively outpatient	11 (55)
Both inpatient/outpatient	9 (45)
Clinic type	
University affiliated	10 (50)
Community based	3 (15)
Operated under Department of Public Health	3 (15)
Private practice	1 (5)
Multiple affiliations	3 (15)

**Table 2:** Primary Care Physicians’ Recommendations for Additional Content and Structure of Hospital Discharge Summaries*

Recommended items	Description	Examples
Actionable to-do-list	Dedicated section in the discharge summary which contains immediate, actionable items which the PCP needs to complete in follow-up	• Laboratories studies needed with a few days of discharge • Pending pathology or culture studies • Referrals to be placed by the PCP
Identification of incidental findings	Create a header to list incidental findings	• Imaging findings with thyroid, lung, or adrenal nodules required follow-up • Lab abnormalities such as new macrocytosis
Justification of medication changes	For medication discontinued include a brief explanation as to the reason for the change	• Explain changes within same drug class (i.e., metoprolol changed to carvedilol) • Why an ACE inhibitor was stopped in a diabetic patient
Duration of medication therapy	Provide detailed timing for antibiotics and high-risk medications	• Antibiotics: dates therapy started and stops, include specific duration for outpatient completion • Anticoagulation: number of weeks/months expected to be on therapy • Opioids: plan for taper vs expectation for refill by PCP
Remove hospital specific care information	Avoid including any plans that would not be relevant for outpatient care	• IV potassium repletion • Daily CBC, transfusion for Hb < 7
Exclude irrelevant details	Provide a summary of care rather than day-to-day changes during the hospitalization	• Avoid: Supplemental oxygen increase to 2 L hospital day 1, then increase to 3 L hospital day 3, then decreased back to 2 L hospital day 4, … • Include: The patient required 3 L of supplemental O2 during hospitalization secondary to pneumonia, but at the time of discharge the patient does not require supplemental oxygen

^*^These items are in addition to the required elements specified by the Joint Commission: reason for hospitalization, significant findings, procedures and treatments provided, patient’s condition at discharge, patient and family instructions, and attending physician signature

### List of Actionable Items Requiring Prompt Follow-up

Most participating PCPs remarked that a brief to-do list at the top of the discharge summary would be helpful to include at the time of hospital discharge. As one participant said, “Just seeing the comments to the physician, which lists everything, all the actionable items, is super useful for me, because then I have a checklist of things to review to make sure that those things [are done] because a primary care visit can get pretty chaotic fast.” This list should contain items such as “outstanding labs results,” “pathology results that are pending,” “culture(s) still pending,” and “referrals [that] have been made/need to be followed up.” Another reiterated the same idea: “… Because there’s such little time [in my primary care visits] … I want to have highlightable, actionable items.” PCPs indicated appreciating when all items requiring prompt attention were in one area, rather than interspersed throughout a long document that they might not have time to read in detail.

### Incidental Findings Requiring Outpatient Follow-up

Many participating PCPs expressed significant anxiety over incidental findings uncovered during a hospitalization that may be unrelated to the reason for hospitalization but require outpatient follow-up. Most of these incidental findings appear on radiological imaging—such as “thyroid nodules,” “lung nodule,” or “nodules on the adrenal gland.” As one participant noted, “I always get scared that something’s going to fall through the cracks like an incidental finding that actually needs to be followed up that nobody picks up and reminds the primary care doctor that it needs to be followed.” Another participant noted that significant findings, which are required by the Joint Commission to be reported in discharge summaries, include incidental findings noting, “significant findings would be anything that would alter treatment or may cause harm because you didn’t address it or follow up whether it’s acutely what is related to [the] hospitalization. And to me incidental findings are still part of future care so significant findings.” This was echoed by another PCP who noted “follow up tests [for] incidental findings … are truly important whether it’s six months down the line [or] twelve months down the line.”

### Justification of Medication Changes

Participants reported frequent difficulty understanding changes in medications during the hospitalization. Several cited medications being changed or discontinued without a clear explanation of why the change occurred. As one interviewee described, “We’ll see this was discontinued … or increased, but there’s not a reason for it and then I’ll have to do a lot more digging.” Participating PCPs specifically highlighted the importance of understanding whether a discontinued medication was contraindicated for future use. Another participant noted, “I see a lot of medications getting changed, but … it’s never in the discharge summary why; like did they have a reaction? or is this medication felt like to be better for the current clinical status? or was it like a mistake? because a lot of times, it'll be like a mistake, it gets left off, but then there's a lot of confusion.” This experience was shared by another participant who said, “A lot of times they’ll stop a med. Let’s say they were on a thyroid med for example. So here’s the admission note shows them on the thyroid med discharge not. You ask the patient why aren’t you on your thyroid med, ‘They told me to hold that’ and of course, the patient doesn’t know why. I asked him did they tell you why, ‘No.’ So, then you have to go what the heck, why did they take them off the med?”.

### Duration of Recommended Medication Use

Participants described difficulty when time periods for medication use were not clearly delineated. For example, “The other kind of medication that gets confused with patients is antibiotics … how long do they take to finish and how many pills that they get. I’ve gotten calls about that, and then I have to go search for it …” Other types of medications that were sources of concern were anticoagulants and controlled substances including opioids and benzodiazepines. One participating PCP noted, “The time course [for anticoagulation] if they know it, it would be nice if they put it in there, so, then the patient and I don’t have to kind of wonder was there some extenuating circumstances for this PE or DVT” and “Plavix is another painful one where you’re like are you supposed to be on it a year or are you supposed to be on a lifetime.” A different participant described patients “getting started on pain medications without clear guidance of like, was this plan[ned] to continue [outpatient]” and “people getting started on benzos for one reason or the other” without it being explained in the discharge summary. Another PCP shared a story of a patient discharged on colchicine with no clarification of the duration and the PCP “had to like dig through all the stuff and figure out, finally, that it was cardiology who recommended that they were on colchicine for three months.”

### General Content Recommendations for Trainees

Several participants observed that trainee discharge summaries were more likely to contain extraneous data. Participants noted that junior trainees often have difficulty concisely summarizing an entire hospitalization and identifying the most pertinent information for the PCP. Participants noted difficulty with a data dump approach that leaves the PCP to “tease out the most important parts” without the full context of the hospitalization and specifically the decision making that led to the choice of tests, consultations, and treatment plans. As one participant said, “paring it down to the essentials that’s the main thing … people they're so scared about not missing [anything] they put everything in and it’s like too much information. And we really just need the pertinent [items] so we don’t get lost in all the note bloat.” Participants reported frustration with discharge summaries that become bogged down in over-detailed information such as day-by-day changes that have become irrelevant by the time of discharge. Additionally, several participants reported concerns about “copy and paste” discharge summaries that include hospital specific plans such as “transfuse for hemoglobin less than seven” or “PRN potassium.” As one participant noted, the inclusion of hospital specific plans discredits the entire goal of the discharge summary. Participants stressed the importance of oversight of discharge summaries and providing trainees with feedback. Some participants requested that senior residents complete all discharge summaries rather than having this important task fall to an intern (or a medical student acting as an intern on the team).

## DISCUSSION

This qualitative study identified ways to expand on current Joint Commission requirements for discharge summaries with specific recommendations that would make the summary more useful to primary care physicians. Participants all spoke to the need for inpatient physicians to “imagine you’re me” and write a narrative as if they themselves were receiving this document in a busy primary care setting. The major components of a desired discharge summary identified by participating PCPs include creation of actionable to-do-lists, identification of incidental findings, description of medication changes, and intended duration of new therapies. Participants further recommended avoiding over-detailed discharge summaries that included every piece of data from the hospitalization, day-to-day hospital details, and copy and paste of notes that contain hospital specific information. It is, however, important to note that some of this extraneous information may be included for billing purposes. But information included solely for billing purposes that is not useful to PCPs, should ideally be minimized, with consideration of revision of existing billing guidelines.

In the creation of actionable to-do-lists participants recommended having a specific section within the discharge summary to highlight and group follow-up items needed immediately after discharge. Those items include recommended outpatient laboratory studies, pending pathology or cultures results, and outpatient referrals which need to be placed by the PCP. Utilizing this approach to group actionable items would help ensure important follow-up items are not missed in the main text of the discharge summary.

Participants recognize that incidental findings may not be tied to the reason for admission, but anything that requires longitudinal attention is relevant to outpatient care and should be clearly noted in a discharge summary. The importance of clear communication about incidental findings is underscored by the prevalence of malignancies identified as incidental imaging findings.^17^ To improve communication about incidental findings, participants suggest discharge summary templates specifically identify any incidental findings which require follow-up outpatient.

The two recommendations related to medication reconciliation include (a) providing a clear rationale for any changes in medication and (b) careful specification of the recommended duration of any new therapies. For medications that are discontinued on discharge, a brief explanation for the reason for the change will assist PCPs to identify when medications may now have a contraindication to be resumed outpatient or perhaps had been inadvertently discontinued. An explanation for switches from one medication to another within the same drug class (i.e., metoprolol changed to carvedilol) is particularly helpful for PCPs who must understand the reasoning for such a change. Clearly noting the dates (rather than just the duration) of inpatient antibiotic therapy and desired additional duration outpatient can eliminate confusion and help PCPs more efficiently and accurately answer patient questions. For patients started on anti-coagulation, understanding both the indication for and time frame of recommended use (e.g., 6 months or indefinite) is helpful for PCPs. For controlled substances, PCPs find it useful to see how many pills a patient has been provided, whether the patient had been advised to taper use, and/or the time frame within which the inpatient team had indicated the patient might expect a refill from their PCP. The importance of accurate and clear documentation of medications at discharge has been highlighted in prior studies which identify 3–64% (median 21%)^18^ of readmissions to be related to medication therapy. This indicates that clear medication documentation can meaningfully improve patient outcomes.

Many study participants received hospital discharge summaries written by trainees and highlighted the importance of providing trainees with targeted feedback on the quality of their discharge summaries. In particular, trainees need guidance to concisely summarize information pertinent to outpatient care and exclude extraneous information and day-to-day details of a hospital stay. Participants recognized that discharge summary composition is a challenging skill which must be taught and requires continuous practice and feedback. Although these recommendations were offered to trainees, they are applicable more broadly to any discharge summary writer.

Limitations of this study include the inclusion of a considerable number of participants from a single academic medical center, although input was also sought from participants practicing in a variety of practice locations. Future studies that include more PCPs in independent practices would be helpful. The participants in the study did describe several items that they deemed to be helpful. Determining if these changes improve primary care physicians’ day-to-day practice will require additional studies. Although the focus of this study was limited to discharge summaries following inpatient medical care, the identified recommended items likely apply to surgical discharge summaries as well; however, this will need to be confirmed.

In conclusion, as healthcare has become more siloed with less direct communication between physicians, improving written discharge summaries remains of great importance. The Joint Commission should consider providing more detailed requirements such as what was recommended by our participants to help improve communication with outpatient providers. Healthcare systems can improve system-wide templates used to write discharge summaries to add prompts/headers to include information that will be useful for the primary care physician. Medical schools and residency programs can enhance trainees’ skills in synthesizing clinical information into a cohesive and concise discharge summary through the addition of curriculum focusing on written communication skills. Together, these changes will hopefully lead to high-quality care following hospital discharge.

### Supplementary Information

Below is the link to the electronic supplementary material.

Supplementary file1 (DOCX 1325 KB)

## Declarations:

### Disclaimer:

The contents of this publication are solely the responsibility of the authors and do not represent the official views of HRSA or the US government.

## References

[1] Auerbach AD, Davis RB, Phillips RS (2001). Physician views on caring for hospitalized patients and the hospitalist model of inpatient care. J Gen Intern Med..

[2] Stevens JP, Nyweide DJ, Maresh S, Hatfield LA, Howell MD, Landon BE (2017). Comparison of Hospital Resource Use and Outcomes Among Hospitalists, Primary Care Physicians, and Other Generalists. JAMA Intern Med..

[3] Wachter RM (2008). The State of Hospital Medicine in 2008. Med Clin N Am..

[4] **Bell CM, Schnipper JL, Auerbach AD, et al.** Association of communication between hospital-based physicians and primary care providers with patient outcomes. J Gen Intern Med. Published online 2009. 10.1007/s11606-008-0882-8. 10.1007/s11606-008-0882-8 PMC2642573 19101774

[5] Oduyebo I, Lehmann CU, Pollack CE (2013). Association of Self-reported Hospital Discharge Handoffs With 30-Day Readmissions. JAMA Intern Med..

[6] **Kripalani S, Jackson AT, Schnipper JL, Coleman EA.** Promoting effective transitions of care at hospital discharge: A review of key issues for hospitalists. J Hosp Med. 2007;2(5). 10.1002/jhm.228. 10.1002/jhm.228 17935242

[7] **Kind AJH, Smith MA.** Documentation of mandated discharge summary components in transitions from acute to subacute care. In: Henriksen K, Battles JB, Keyes MA, Grady ML, editors. Advances in Patient Safety: New Directions and Alternative Approaches (Vol. 2: Culture and Redesign). Rockville (MD): Agency for Healthcare Research and Quality (US); 2008 Aug. 21249900

[8] Robelia PM, Kashiwagi DT, Jenkins SM, Newman JS, Sorita A (2017). Information Transfer and the Hospital Discharge Summary: National Primary Care Provider Perspectives of Challenges and Opportunities. J Am Board Fam Med..

[9] Polyzotis PA, Suskin N, Unsworth K (2013). Primary Care Provider Receipt of Cardiac Rehabilitation Discharge Summaries. Circ Cardiovasc Qual Outcomes..

[10] **Robelia P, Kashiwagi D, Newman J, Sorita A, Jenkins S.** Information transfer and the hospital discharge summary: National primary care provider perspectives of challenges and opportunities. J Am Board Fam Med. 2017;30(6). 10.3122/jabfm.2017.06.170194. 10.3122/jabfm.2017.06.170194 29180550

[11] **Sorita A, Robelia PM, Kattel SB, et al.** The Ideal Hospital Discharge Summary: A Survey of U.S. Physicians. J Patient Saf. 2021;17(7). 10.1097/PTS.0000000000000421. 10.1097/PTS.0000000000000421 28885382

[12] Silver AM, Goodman LA, Chadha R (2022). Optimizing Discharge Summaries: A Multispecialty, Multicenter Survey of Primary Care Clinicians. J Patient Saf..

[13] Bradley EH, Curry LA, Devers KJ (2007). Qualitative Data Analysis for Health Services Research: Developing Taxonomy, Themes, and Theory. Health Serv Res..

[14] **Nelson J.** Using conceptual depth criteria: addressing the challenge of reaching saturation in qualitative research. Qual Res. 2017;17(5). 10.1177/1468794116679873.

[15] **Charmaz K.** Premises, principles, and practices in qualitative research: Revisiting the foundations. Qual Health Res. 2004;14. 10.1177/1049732304266795. 10.1177/1049732304266795 15296667

[16] **Hsieh HF, Shannon SE.** Three approaches to qualitative content analysis. Qual Health Res. 2005;15(9). 10.1177/1049732305276687. 10.1177/1049732305276687 16204405

[17] O’Sullivan JW, Muntinga T, Grigg S, Ioannidis JPA (2018). Prevalence and outcomes of incidental imaging findings: umbrella review. BMJ..

[18] **El Morabet N, Uitvlugt EB, van den Bemt BJF, van den Bemt PMLA, Janssen MJA, Karapinar-Çarkit F.** Prevalence and Preventability of Drug-Related Hospital Readmissions: A Systematic Review. J Am Geriatr Soc. 2018;66(3). 10.1111/jgs.15244. 10.1111/jgs.15244 29468640

