# Supplementary material for: Primary Care Physicians’ Perspectives on High-Quality Discharge Summaries
Source: J Gen Intern Med. 2023 Nov 27;39(8):1438–43. doi: 10.1007/s11606-023-08541-5 (PMC11169121; doi:10.1007/s11606-023-08541-5)

**Appendix 1**

**Interview Guide:** Physician Perspectives on Discharge Summaries

**Purpose:** To learn about outpatient physician (family medicine and internal medicine) perspectives about what makes a high-quality discharge summary

**Introduction:** *Thank you for agreeing to be a part of this study. What we hope to gain with this study is the perspective of primary care providers on hospital discharge summaries. We hope that this information will inform other providers about what content and structure of a discharge summary is most helpful for primary care providers who are caring for recently discharged patients. As a reminder, being part of a research study is completely voluntary and you may stop this interview at any point. I trust that you have reviewed and completed the consent form. Are there any questions I can answer regarding the study before we get started?*

**GENERAL IMPRESSION OF HOSPITAL DISCHARGE SUMMARIES**

- To start out, can you tell me a little bit about how you would describe your clinical practice?

- *Can you start by telling me about what you think is discharge summary is for?*
- *How do you use a discharge summary, personally? For what purpose and in what way?*
- *Do you read the entirety of the discharge summary or parts of it?*
- *If only parts, which ones and why?*
- *What do you read first?*
- *Why?*
- *When do you read it (before seeing the patient, during the visit, or after the visit)? And, in which circumstances? (i.e. Do you only read it if you cannot figure out information from the patient?)*
- *Do you read all summaries for every patient that has been recently discharged?*
- *If not, how do you decide which summaries to read and which not to read?*
- *Do you ever look for other information in the hospital record?*
- *If so, what and in which circumstances?*

- *Can you tell me in general your overall impression of the current hospital discharge summaries that you have received?*
- *After only reading the discharge summary, do you feel that you have all the information needed to seamlessly resume care of the patient?*
- *If not, what is usually missing?*

- *Can you share with me any stories about your experiences with patients related to*
- *A good discharge summary that allowed for you to have a great hospital discharge visit with a patient?*
- *A bad discharge summary that got you or one of your patients into trouble? Or resulted in a readmission?*
- *Your experience with how NOT having access to a discharge summary when you needed it impacted*
- *Your care of a patient*
- *Your relationship with a patient/your patient’s trust in you as a clinician*
- *How often do you estimate you have access to a DC summary in advance*

**RELATIONSHIP WITH CLINIC/HOSPITAL**

- *How would you describe your clinic’s relationship with the health systems that you work with?*
- *Is it a close relationship?*
- *Do you have contacts there?*
- *Do you have meetings with those health systems to help coordinate care?*

- How do you get notified your patients have been admitted?
- What do you do when you get this notification?
- Are there things that you as a PCP think it would be helpful to share with inpatient colleagues when your patients are admitted that may not be clear just from reading the patients chart?

**PATIENT SAFETY**

- *Have you or any of your colleagues had any experiences where information was omitted from the hospital discharge summary that directly affected the care of that patient?*
- *If so, did you provide feedback to the doctor who wrote the discharge summary? And if so, how?*

- *If you have a question regarding a discharge summary your received how do you address it?*
- Probe *– do you review the electronic health record, call/message the physician who discharged the patient?*

- *What has been your experience when you’ve tried to get more information about a  patient's hospital stay in the past?*

**STRUCTURE**

- *Do you have a preferred formatting of the hospital course/hospital problems?*
- Probe – *do you prefer paragraph narratives or bullet points? Why?*

- *Comparing these 4 discharge summaries templates* (shows templates to participant) *do you have a preference regarding the order of items?*

- *Is there anything that is important to you to know about your patient’s hospital course that is missing from these templates?*

**CONTENT**

- *What do you think the essential components are of a discharge summary?*

- *The joint commission requires the following components within the discharge summary: (1)reason for hospitalization, (2) significant findings, (3) procedures and treatment provided, (4) patient’s discharge condition, (5) patient/family instructions, and (6) attending physician’s signature.*
- *What do you think about these essential components?*

- *Out of the discharge summaries that you received in the last month; what percentage contain all 6 of these components that the joint commission requires?*

- *You will notice that all 4 discharge templates (*show discharge summary templates) *have these required components, but each have additional components as well.*

- *Do you notice any specific components that you think would be helpful to receive on all your discharge summaries?*
- Probe *– for example, one of the templates noted the patient’s code status during the hospitalization, do you find that helpful?*

- *Are there components that you wish were included on all discharge summaries?*
- *Any components you wish were omitted to save you time in reviewing the document?*

**Appendix 2**

Examples of existing electronic health record discharge summary templates from different health systems that include the items specified by the Joint Commission (reason for hospitalization, significant findings, procedures and treatments provided, patient’s condition at discharge, patient and family instructions, and attending physician signature) with variable additional material.


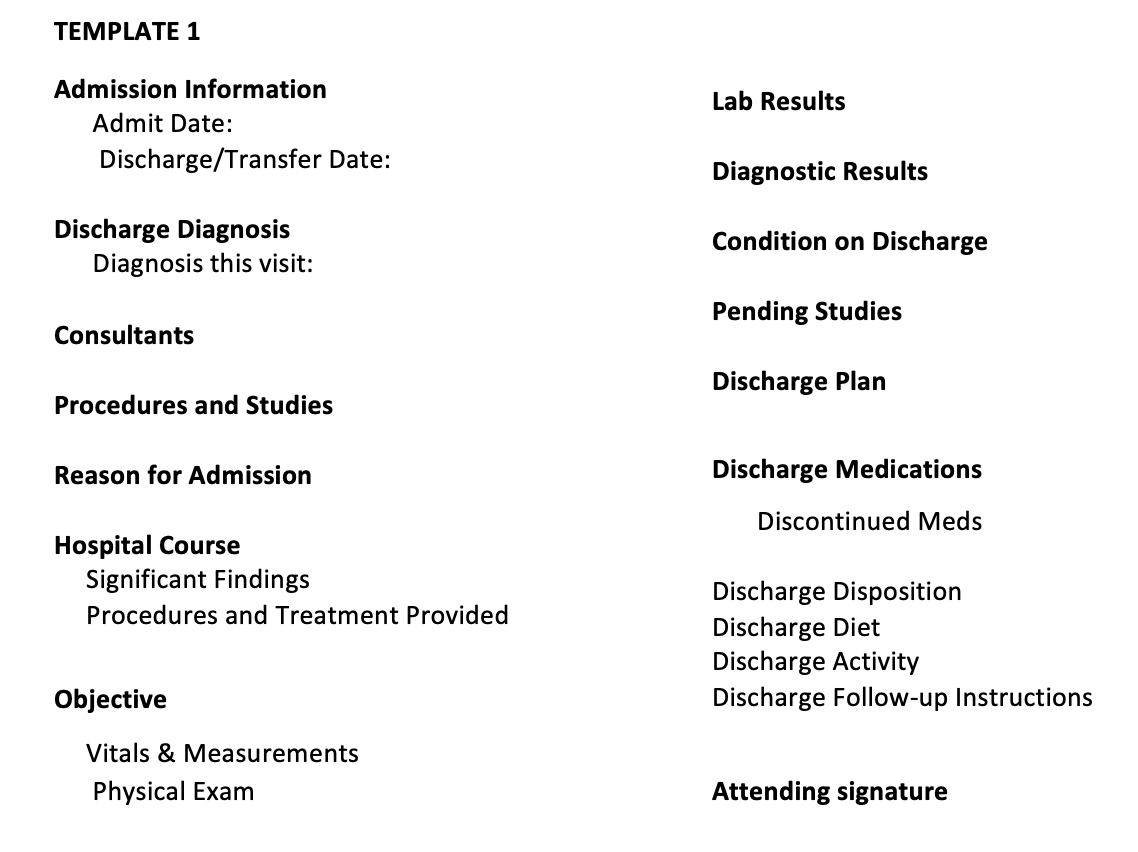


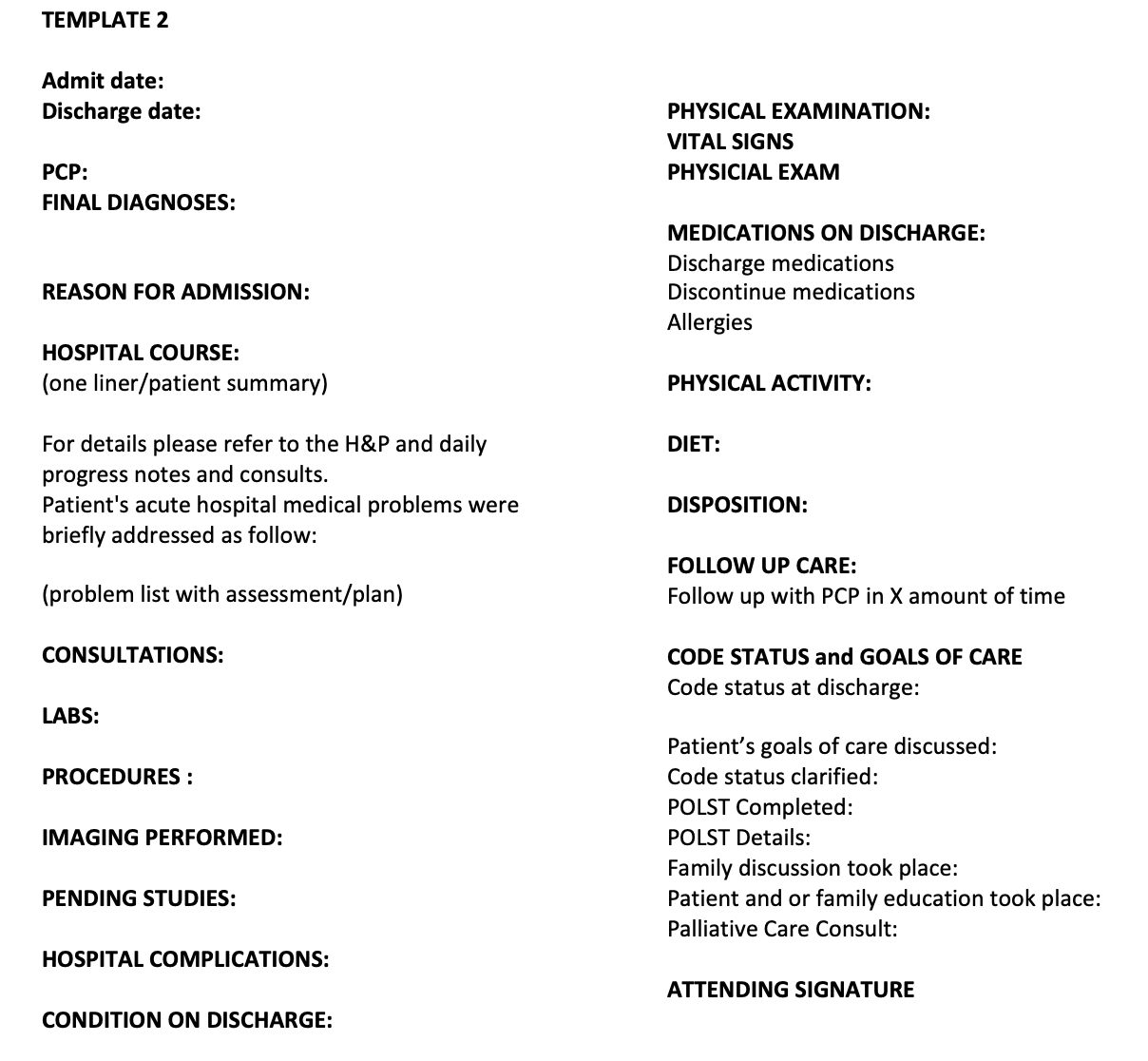


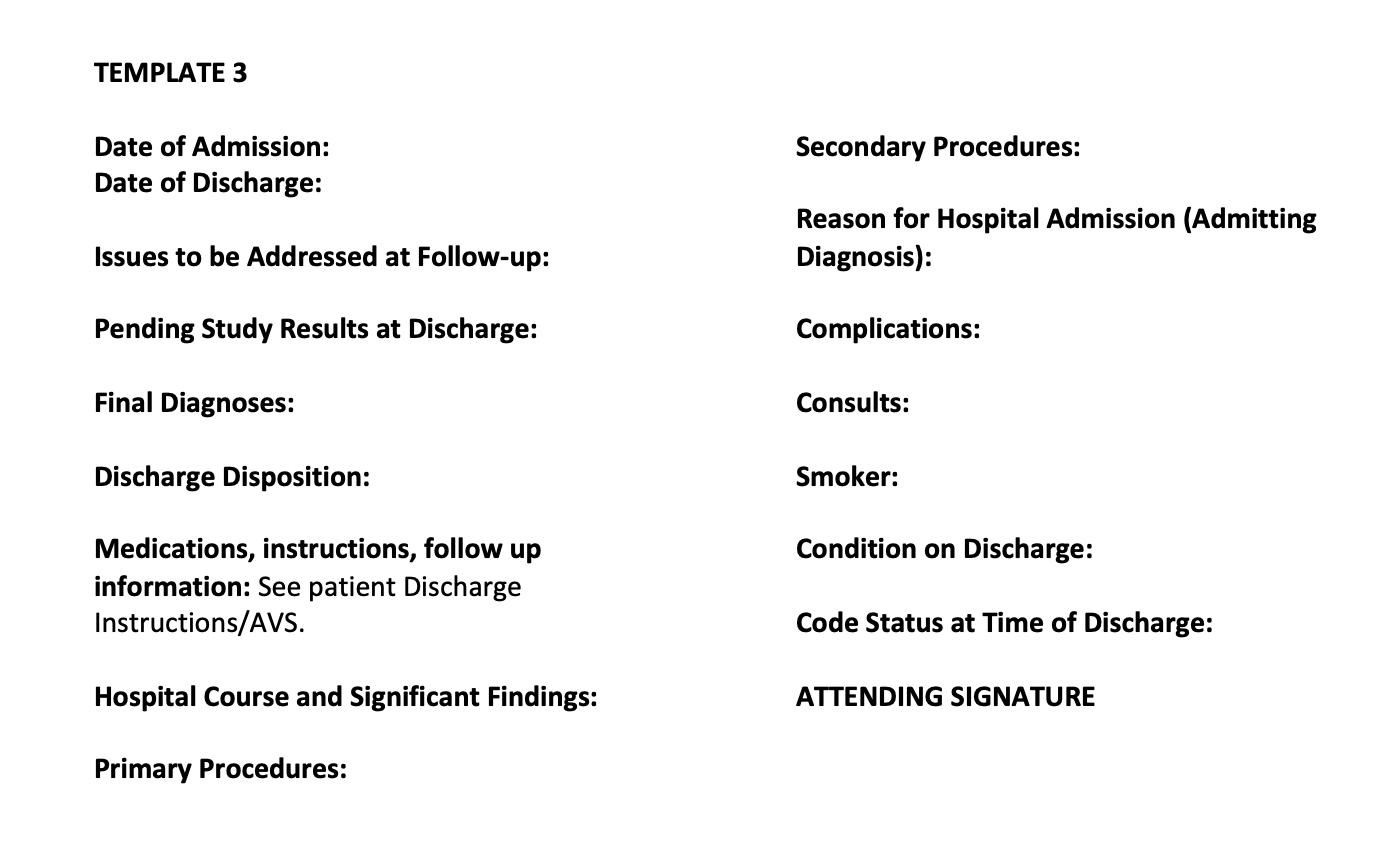


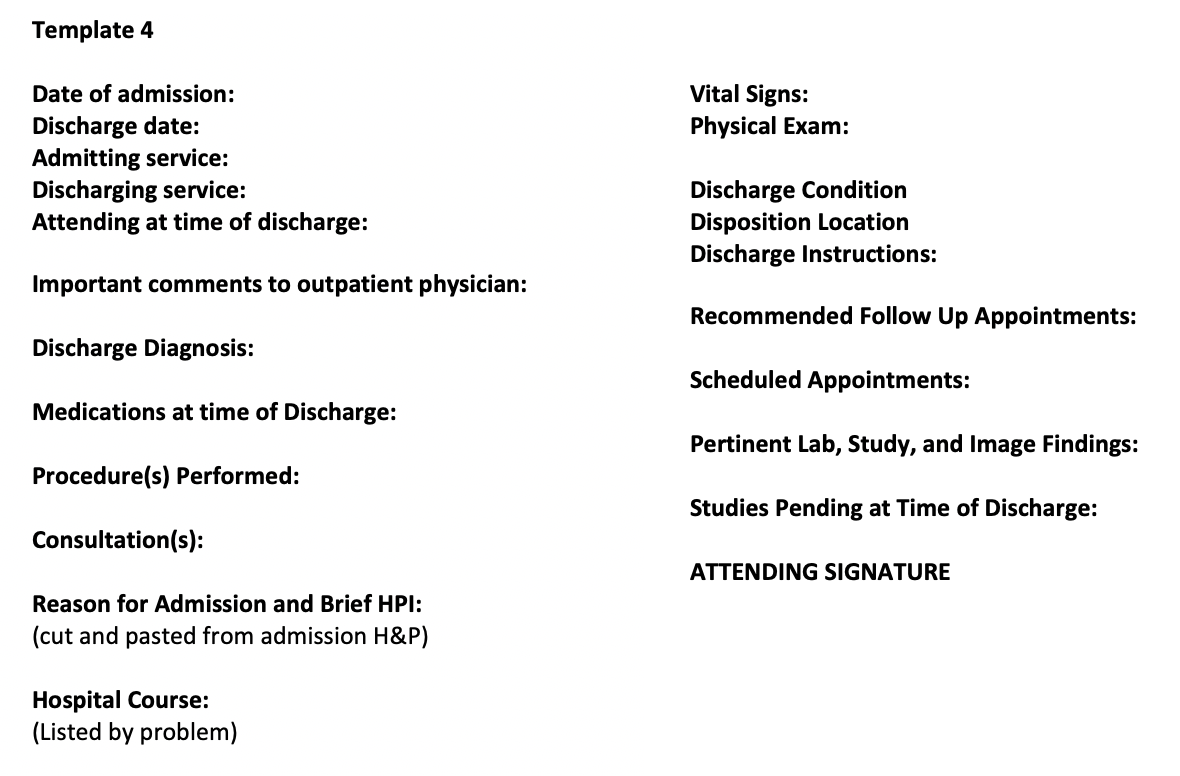

Supplement: Supplementary file 1 — Supplementary file1 (DOCX 1325 KB) [file 11606_2023_8541_MOESM1_ESM.docx]
